# Supplementary material for: Gut microbiota composition in colorectal cancer patients is genetically regulated
Source: Sci Rep. 2022 Jul 6;12:11424. doi: 10.1038/s41598-022-15230-6 (PMC9259655; doi:10.1038/s41598-022-15230-6)
Supplement: Supplementary file 1 — Supplementary Information 1. [file 41598_2022_15230_MOESM1_ESM.docx]

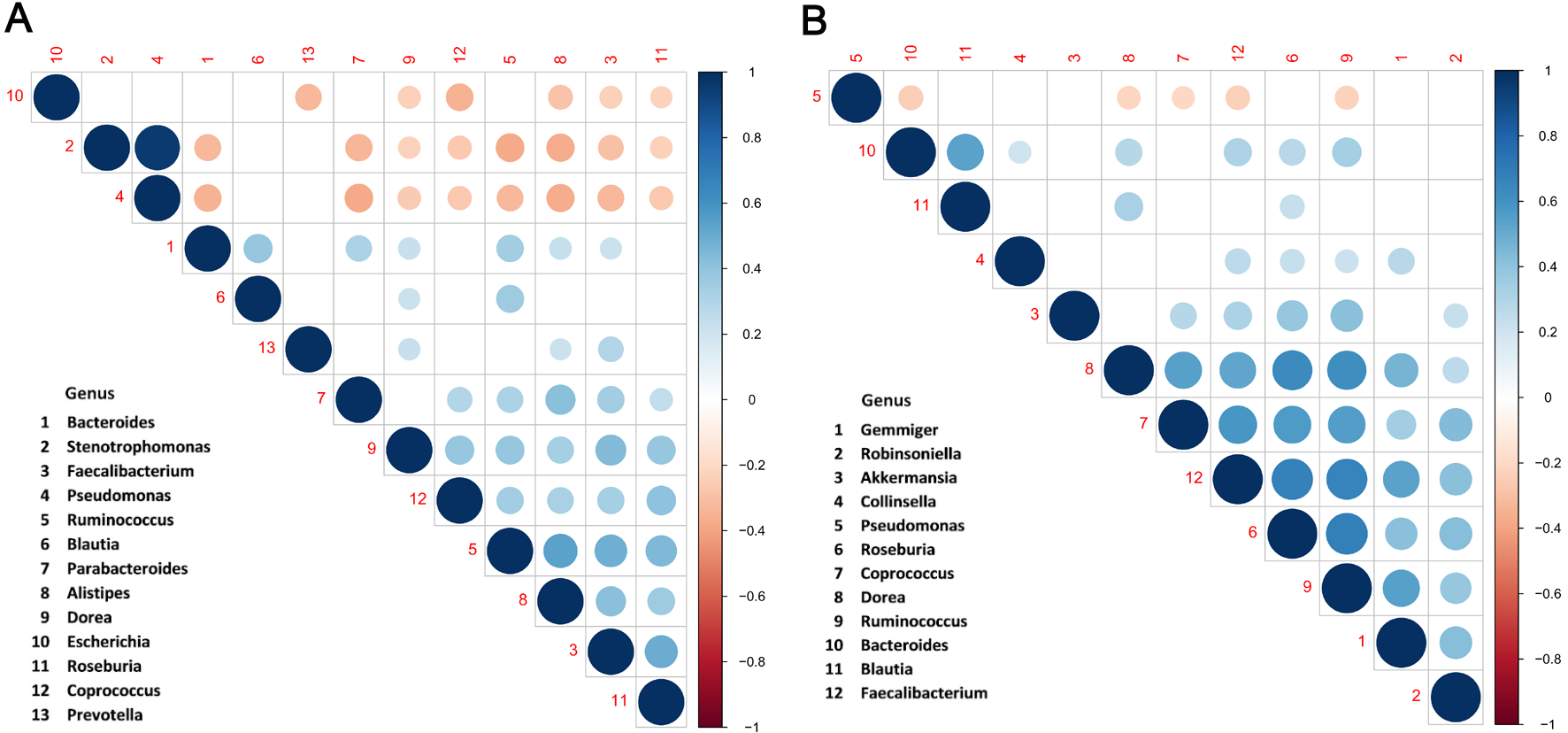


**S1 Fig. Correlation matrices for OTU abundance.** A) V1-V2-V3 dataset. B) V4-V5-V6 dataset. Numbers along the matrix indicate the OTU in the list. Pearson's correlation coefficients are indicated on a dichromatic scale, and the sizes of the circles are proportional to the correlation coefficient.
